# Preparation of Hollow Flower-Like Microspherical β-Bi_2_O_3_/BiOCl Heterojunction and High Photocatalytic Property for Tetracycline Hydrochloride Degradation

**DOI:** 10.3390/nano10010057

**Published:** 2019-12-25

**Authors:** Shulin Kong, Zhaohui An, Wenwen Zhang, Zhihao An, Ming Yuan, Donghui Chen

**Affiliations:** 1 College of Chemical and Environmental Engineering, Shanghai Institute of Technology, Shanghai 201418, China; kongslsit@163.com (S.K.); sit1529631543@163.com (Z.A.); ym13635651450@163.com (M.Y.); 2 College of Environmental Science and Engineering, Donghua University, Shanghai 201620, China; 15921789816@163.com; 3 Institute of Foreign Languages, Shanghai DianJi University, Shanghai 201306, China

**Keywords:** β-Bi_2_O_3_/BiOCl, hollow flower-like microsphere, photocatalytic activity, heterojunction, tetracycline

## Abstract

Tetracycline cannot be effectively degraded in wastewater treatment. Therefore, the development of excellent photocatalysts is of significant importance for environmental protection. In this study, a β-Bi_2_O_3_/BiOCl heterojunction photocatalyst with hollow flower-like microspheres was successfully synthesized by the in-situ reaction of HCl and β-Bi_2_O_3_ hollow spheres. The prepared samples are characterized by Scanning electron microscopy, Transmission electron microscopy, X-ray diffraction, X-ray photoelectron spectroscopy, N_2_ physical adsorption, UV-vis diffuse reflectance spectroscopy, and Photoluminescence. Then, research on the photocatalytic performance for the degradation of tetracycline hydrochloride was conducted. The results show that the photocatalytic performance of the β-Bi_2_O_3_/BiOCl composite is significantly better than the β-Bi_2_O_3_ and BiOCl. The increase in photocatalytic activity is due to the formation of a heterojunction between β-Bi_2_O_3_ and BiOCl, which effectively promotes the separation of photogenerated electron-hole pairs. Additionally, the heterojunction nanocomposite demonstrated the outstanding photocatalytic stability after five cycles, which indicates that the material can be used for water environment purification. This paper provides assistance for studying the photocatalytic mechanism of heterojunction photocatalytic materials.

## 1. Introduction

In recent years, with the rapid development and application of new environmental analysis technologies, the focus of environmental workers on environmental pollution and human health has gradually extended from traditional typical pollutants to trace pollutants in the environment, such as antibiotics [1,2,3]. China is not only a big country in antibiotic production but also a big country in antibiotic use. The annual production and consumption of antibiotics are dozens of times that of foreign countries [4]. At present, the abuse of antibiotics has caused it to enter our soil and water in various ways, such as the medical industry wastewater, animal feces, and so on. Antibiotics have had a serious impact on the environmental problems of our country [5,6,7]. When the antibiotic wastewater enters the water body, as an external factor, it will break the original biological chain, damage the environment, and endanger people’s health [8,9]. Tetracycline hydrochloride (TC) is one of the most typical antibiotics. At present, TC has been detected in surface water, groundwater, lake bottom sludge, and even drinking water, mainly from industrial pharmaceutical wastewater, hospital wastewater, and aquaculture wastewater [10,11]. These trace amounts of antibiotics entering the water not only cause toxicity to aquatic organisms but also stimulate the resistance of pathogenic microorganisms, which has a huge potential impact on the entire ecosystem [12]. However, traditional sewage treatment technology cannot effectively remove tetracycline from water [13]. The existing methods of removing antibiotic pollutants mainly include sludge adsorption, chemical oxidation, membrane separation, and microbial degradation [14]. TC antibiotic wastewater is a kind of high-concentration organic medical wastewater containing refractory organic compounds and biotoxic substances, with a large change in chemical oxygen demand concentration and poor biodegradability. Conventional activated sludge method and other water treatment technologies have little effect on the treatment of antibiotic wastewater [15,16]. Therefore, the treatment of TC antibiotic wastewater is very important for the comprehensive treatment of the water environment in China, and the development of effective treatment methods has great social value and environmental benefits.

Photocatalysis is an effective advanced oxidation method, which is widely used due to its advantages of simple operation, low energy consumption, wide application range, avoiding secondary pollution, and high treatment efficiency [17,18]. The photocatalytic process can produce strong oxidation free radicals, which can effectively remove organic compounds in water. The results show that photocatalysis is an effective method to remove antibiotics from water [16,19]. Zhu et al. [20] irradiated tetracycline solution with ultraviolet light catalyzed by nano TiO_2_, and the degradation rate of tetracycline reached 95% after irradiation for 60 min. However, traditional photocatalyst TiO_2_ can only absorb and utilize ultraviolet light, and cannot effectively utilize sunlight (ultraviolet light only accounts for 5% of the solar spectrum). Meanwhile, its low photonic quantum efficiency limits its practical application [21,22]. Therefore, it is important to develop photocatalytic materials with the visible light response.

In recent years, bismuth photocatalysts (e.g., Bi_2_O_3_, BiOCl, BiOBr, BiOI, BiVO_4_) have become one of the research hotspots in the field of photocatalysis. As a kind of bismuth photocatalyst, Bi_2_O_3_ has many crystal types and strong visible light absorption ability, so it should be widely used in the field of photocatalysis [23]. Compared with traditional photocatalyst TiO_2_, Bi_2_O_3_ has a narrow band gap (about 2.8 eV), a large number of oxygen vacancies, and a good optical conductivity. Therefore, it has a higher utilization rate of sunlight and a stronger oxidation capacity [24,25,26]. There are two main crystal phases in Bi_2_O_3_, the bandgap of monoclinal phase α-Bi_2_O_3_ is about 2.8 eV and the bandgap of the quartet crystal phase β-Bi_2_O_3_ is about 2.4 eV, so β-Bi_2_O_3_ has stronger visible light response-ability [27,28,29,30]. However, there are two major defects when using β-Bi_2_O_3_ as photocatalyst alone—one is that photogenerated electrons and photogenerated holes are easy to compound, and the quantum efficiency is low. The second is that β-Bi_2_O_3_ is unstable in the reaction process when the temperature is slightly changed, phase transformation will occur, which will lead to the formation of (Bi_2_O_2_)CO_3_ [31]. Therefore, relatively few studies have used β-Bi_2_O_3_ alone. As a photocatalytic semiconductor material with good photocatalytic activity, BiOCl has received great attention from researchers in recent years [32,33]. BiOCl is an indirect bandgap semiconductor with a highly anisotropic layered structure, good chemical stability and adsorption capacity, and bandgap matching with Bi_2_O_3_, which makes it have high photocatalytic activity. BiOCl is better than TiO_2_ in the degradation of methyl orange under ultraviolet light [22]. The band gap of BiOCl is between 3.2 and 3.5 eV, and the visible light response is poor compared with β-Bi_2_O_3_. In view of the low photocatalytic efficiency of monomers BiOCl and β-Bi_2_O_3_, the advantages of the two semiconductors can be comprehensively utilized when they are combined into heterogeneous structures. The combination of the two semiconductors to form heterogeneous junctions will theoretically improve the adsorption effect of the catalyst and facilitate the subsequent photocatalytic reaction. In addition, heterojunction will promote the separation of photogenerated electrons and holes, and improve the photocatalytic performance of β-Bi_2_O_3_ [34,35]. Many studies on the preparation of β-Bi_2_O_3_ and BiOCl composites have proved that the combination of the two can improve the photocatalytic properties of β-Bi_2_O_3_ [36,37]. However, few studies have been conducted on the photocatalytic performance of β-Bi_2_O_3_/BiOCl heterojunction in visible light, especially on the degradation of tetracycline by β-Bi_2_O_3_/BiOCl heterojunction with hollow flower sphere morphology in visible light has not been reported. Therefore, in this study, hollow, spherical β-Bi_2_O_3_ was synthesized by the solvent thermal method and hollow flower sphere morphology β-Bi_2_O_3_/BiOCl heterojunctions were synthesized by the ultrasound-assisted hydrochloric acid impregnation method. XRD, SEM, XPS, BET, and UV-vis were used to characterize the structure, morphology, composition, specific surface area, and optical properties of the samples. The band structure of the β-Bi_2_O_3_/BiOCl heterojunction is calculated by using the UV-vis test analysis. The tetracycline (TC) was used as the target degradant to test the photocatalytic degradation performance of the sample under visible light irradiation, and the effects of β-Bi_2_O_3_/BiOCl composites prepared by different molar ratios n (β-Bi_2_O_3_)/n (BiOCl) on the photocatalytic activity were investigated. The photocatalytic activity test shows that the performance of the β-Bi_2_O_3_/BiOCl composite photocatalyst is obviously better than that of the monomer β-Bi_2_O_3_. Lastly, we also proposed a possible visible-light photodegradation mechanism of tetracycline (TC) over the β-Bi_2_O_3_/BiOCl heterojunction photocatalyst. 

## 2. Materials and Experimental 

### 2.1. Materials

Materials were purchased from Shanghai Titan Scientific Co., Ltd. (Shanghai, China): bismuth(III) nitrate pentahydrate (Bi(NO_3_)_3_·5H_2_O, AR, 99%), hydrochloric acid (HCl, 36–38%), ethanol (C_2_H_5_OH, AR, 99.7%), Glycerol (C_3_H_8_O_3_, AR, 99%). Tetracycline hydrochloride (TC, 99%) was purchased from Adamas Reagent Co., Ltd. (Shanghai, China). Deionized water (DI) supplied by our university was used in the experiments. All chemicals were used directly and without further purification. 

### 2.2. Synthesis of β-Bi_2_O_3_/BiOCl

#### 2.2.1. Preparation of β-Bi_2_O_3_

β-Bi_2_O_3_ hollow microspheres were synthesized by a simple template-free hydrothermal method followed by thermal treatment based on Yan’s work with minor modifications [38]. Firstly, 6 mmol of Bi(NO_3_)_3_·5H_2_O was dissolved in a mixed solution that contained glycerol (30 mL) and absolute ethanol (30 mL) by ultrasonic dispersion and vigorous stirring for 30 min. Subsequently, the mixed solution was transferred into a 100 ml stainless steel autoclave (Lined with polytetrafluoroethylene), then put it into the air-blast constant temperature drying box, heated at 160 ℃ for 3 h. The reactor was naturally cooled to room temperature, the obtained precipitation was washed with deionized water, and absolute ethanol for three times, respectively, followed by drying at 70 ℃ for 10 h. The dried product was calcined at 270 ℃ for 2 h in a muffle furnace at a heating rate of 2 ℃/min. The product was obtained after cooling to room temperature.

#### 2.2.2. Preparation of β-Bi_2_O_3_/BiOCl

The β-Bi_2_O_3_/BiOCl heterojunction photocatalyst was prepared by an in-situ treatment of β-Bi_2_O_3_ with HCl. Firstly, 0.5 g of as-prepared β-Bi_2_O_3_ was dispersed in 50 mL deionized water by ultrasonic treating. Subsequently, a certain amount of 0.5 mol/L HCl solution was dropwise added with vigorously stirring for 30 min at 50 ℃ After treatment with HCl; the precipitate was recovered by centrifugation, the collected precipitate was washed several times with deionized water and ethanol, then put it in a blast drying box and dry it at 70 ℃ for 10 h. By changing the amount of HCl (1.075 mL, 2.15 mL, 3.225 mL, 3.87 mL) added in the above synthesis process, a series of β-Bi_2_O_3_/BiOCl heterojunction photocatalysts with 40%, 66.7%, 85.7% and 94.7% (These are the molar contents of BiOCl) amount of BiOCl were prepared respectively. Marked as BC-0.5, BC-1, BC-1.5, and BC-1.8, respectively. By following this procedure, the pure BiOCl was prepared in the presence of HCl in excess.

### 2.3. Sample Characterization

The crystal structure and phase of the as-obtained sample were identified by a PANalytical X’ Pert PRO X-ray diffractometer (Osaka, Japan) with Cu Kα (λ = 0.15418 nm) incident radiation. The X-ray diffraction (XRD) patterns were obtained for 2*θ* angles from 10° to 90° at a scan rate of 0.05° min^−1^. Materials were characterized by a HITACHI S-3400 N scanning electron microscope (SEM, Tokyo, Japan) at an accelerating voltage of 15 kV to research the surface morphology. The distributions of the elements of the samples were captured by a tracing electron microscope-energy spectrometer (SEM-EDS) (SEM: JSM-5600 LV, JEOL, Tokyo, Japan; EDS: IE 300X, Oxford, UK). High-resolution transmission images (HRTEM) were taken using a JEOL JEM-2100F electron microscope (Tokyo, Japan). The surface element composition and chemical state of the prepared sample were characterized by X-ray photoelectron spectroscopy (XPS, Al Kα, hv = 1486.71 eV, Waltham, MA, USA). Peak positions in all of the XPS spectra were calibrated with C 1 s at 284.6 eV as the reference level. UV-vis diffuse reflectance spectroscopy (UV-3600, Shimadzu, Kyoto, Japan) was used to examine the photophysical properties of the samples and the range of scanning was 200–800 nm. BaSO_4_ was used as the reflectance standard. The photocatalytic activity of samples was determined by Beijing Purkinje General Instrument production of TU-1900 at the wavelength of 356 nm. BET surface areas of the samples were measured with a surface area and porosimeter analyzer (ASAP 2460, Shanghai, China). The fluorescence characteristics were measured on a fluorescence spectrometer (FP-6500, Tokyo, Japan) with an excitation wavelength set to 340 nm. The photo-electrochemical properties of the samples were measured on an electrochemical workstation (CHI 660E, Shanghai, China). The Xe lamp is used as the light source, Ag/AgCl is the reference electrode, Pt is the auxiliary electrode, and the FTO glass coated with the sample is the working electrode.

### 2.4. Photocatalytic Performance Tests

The photocatalytic performance of β-Bi_2_O_3_/BiOCl heterojunction photocatalysts was evaluated by the photodegradation rate of TC solution in a photochemical reactor (China Education Au-light Co., Ltd., Beijing, China), we are equipped with 300 W Xe lamp as the source of visible light, and the UV light was filtered through a 400-nm filter. Specific steps are as follows: we added 100 mL of TC solution (20 mg/L) to the quartz reactor of the photocatalytic experimental device, add 20 mg of the prepared sample under constant magnetic stirring, which was about 10 cm from the liquid surface of the suspensions to the lamp. Before turning on the light source, we kept the light-proof environment and stirred for 30 min to achieve an equilibrium state of adsorption-desorption between the photocatalyst and the tetracycline molecule. After dark processing, we turned on the light source for photocatalytic experiments. Every 20 min, we took out 5 mL of the reaction solution, centrifuged in a centrifuge for 3 min, and took out the supernatant for testing. The absorbance of the supernatant was measured by a UV-visible spectrophotometer at 356 nm. The photocatalytic degradation rate (*W*/%) was calculated according to the following Equation (1):

*W* = (*Co* − *Ct*)/*Co* × 100% = (*Ao* − *At*)/*Ao* × 100% (1)

In this formula, *Co* and *Ct* are the concentration of TC solution before lighting on and at the time of *t* after photodegrading. *Ao* and *At* are the absorbance of TC solution before light up and at the time of *t* after photodegradation, respectively.

A pseudo-first-order kinetic model was used to evaluate the photocatalytic activity, and the degradation kinetics constant k was obtained as Equation (2):

*ln*(*Co*/*Ct*) = *kt* (2)

In this formula, *Co*, *Ct*, and *t* represent the concentration of contaminant solution before illumination, the concentration of pollutant solution when Xe lamp irradiation time is *t*, and irradiation time, respectively.

### 2.5. Recycling Performance of the Sample

After photocatalytic degradation, the photocatalyst with the adsorbed TC was collected by auto-precipitation and centrifugation, washed several times by using C_2_H_5_OH and DI as the adsorbent to regenerate. Then reused for the next time of photocatalytic degradation to evaluate the photocatalytic stability and reusability of the β-Bi_2_O_3_/BiOCl heterojunction photocatalyst.

## 3. Results and Discussion

### 3.1. Phase Composition and Microstructure Analysis of the Samples

**Figure 1:**
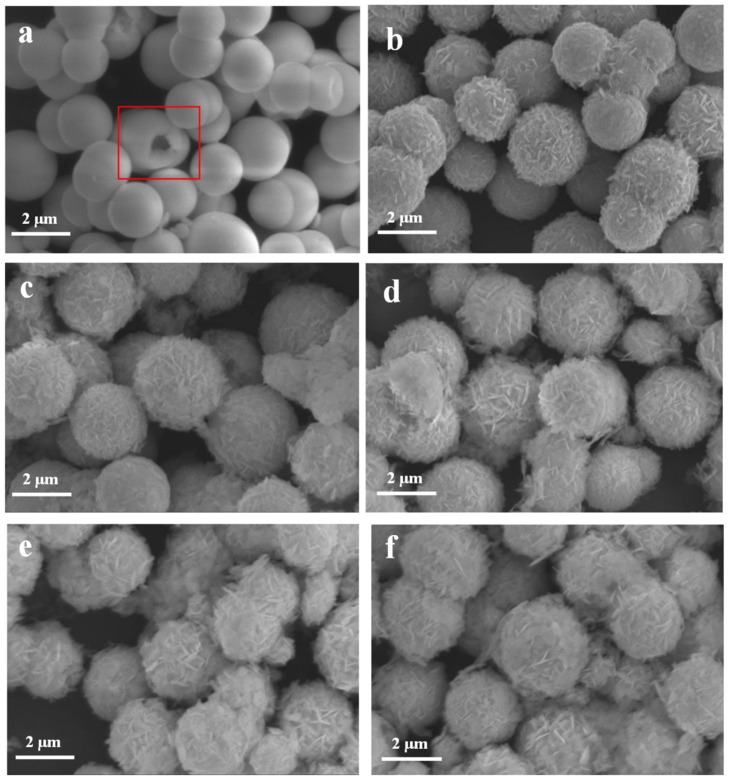
SEM images of Bi_2_O_3_ (**a**), BC-0.5 (**b**), BC-1 (**c**), BC-1.5 (**d**), BC-1.8 (**e**), and pure BiOCl (**f**).

In order to characterize the micromorphology and structure of samples, SEM-EDS and HRTEM were used. Figure 1a is the image of pure Bi_2_O_3_, compared with the images of β-Bi_2_O_3_/BiOCl (Figure 1b–e) and pure BiOCl (Figure 1f), it is easy to find that the prepared β-Bi_2_O_3_ has a microsphere appearance, the size is about 2 µm, the surface is relatively smooth, and the size is relatively uniform. It can be seen from the broken microspheres in the red box of Figure 1a that the prepared β-Bi_2_O_3_ has a hollow structure. This unique hollow spherical structure allows the photocatalytic semiconductor to have a higher specific surface area, which is advantageous for the adsorption amount and photocatalytic activity of the sample. The SEM images of the β-Bi_2_O_3_/BiOCl heterojunction are shown in Figure 1b–e and show that BiOCl nanosheets are tightly covered on the β-Bi_2_O_3_ surface. As the concentration of HCl increases, more BiOCl nanosheets are formed on the β-Bi_2_O_3_ surface. When the HCl concentration is low, it can be seen from Figure 1b–d that most BiOCl nanosheets grow upright on the β-Bi_2_O_3_ surface. However, when the HCl concentration continues to increase, it can be seen from Figure 1e,f that due to the large increase in BiOCl nanosheets, many BiOCl nanosheets are stacked together and even cover the β-Bi_2_O_3_ surface, this greatly affects the photocatalytic properties of the material. It can be inferred that the BiOCl nanosheet is gradually grown on the surface of the β-Bi_2_O_3_ hollow sphere by a chemical etching reaction.

**Figure 2:**
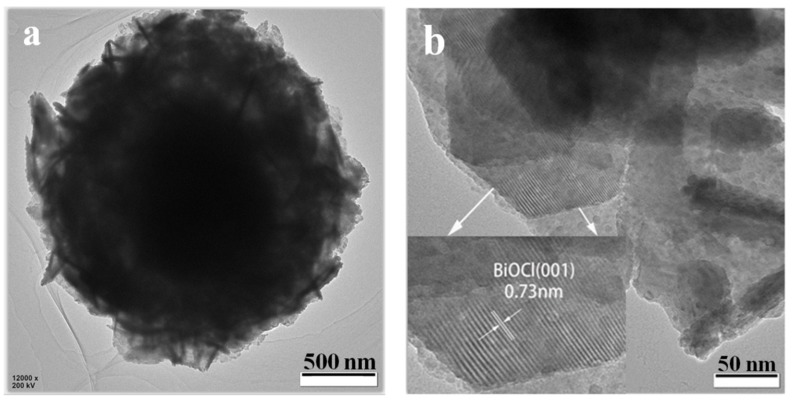

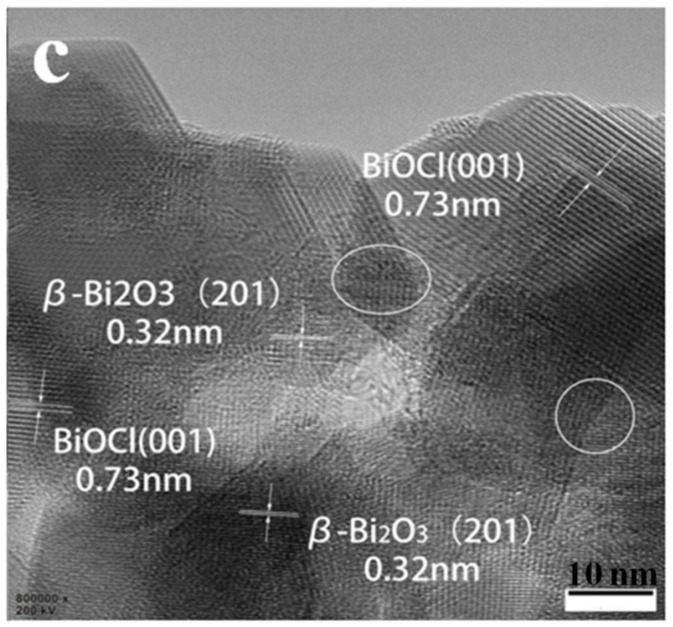
TEM image of BC-1.5 (**a**) and high-resolution TEM images for the surface (**b**), and inner (**c**), parts of the BC-1.5.

In order to determine the detailed structural information, BC-1.5 was further characterized by low-magnification TEM and high-resolution TEM (HRTEM) analysis. As shown in Figure 2, the TEM image in Figure 2a reveals that the BC-1.5 sample is a flower ball of about 2 µm in size; many nanosheets grow on the surface of the ball; this is consistent with the characterization of SEM. The HRTEM image in Figure 2b shows that the surface nanosheets have clear lattice fringes with a lattice spacing of approximately 0.73 nm, corresponding to the (001) planes of tetragonal phase BiOCl [39]. The HRTEM image in Figure 2c for the inner parts of the BC-1.5 reveals that two types of lattice fringes. The interlayer distance of 0.73 nm corresponds to the (001) lattice plane of BiOCl, and that of 0.32 nm is consistent with the (201) plane of β-Bi_2_O_3_ [40,41]. It can be seen from the white circles that there are some mixed lattice fringes. This shows that β-Bi_2_O_3_ grains and BiOCl matrix are embedded in each other inside the material. Therefore, based on the above results, it is inferred that the β-Bi_2_O_3_/BiOCl heterojunction is formed by in situ growth of BiOCl nanosheets and superposition on the surface of the β-Bi_2_O_3_ hollow sphere.

**Figure 3:**
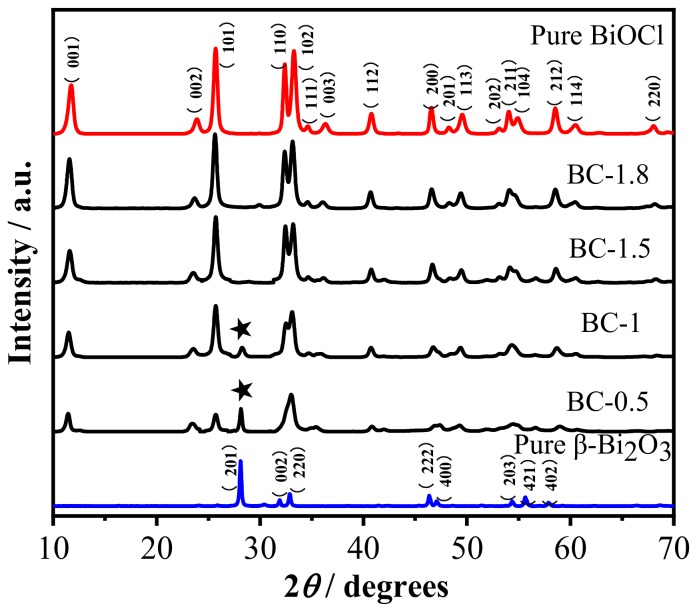
XRD patterns of β-Bi_2_O_3_, BiOCl, and β-Bi_2_O_3_/BiOCl heterojunctions with different proportions.

**Figure 4:**
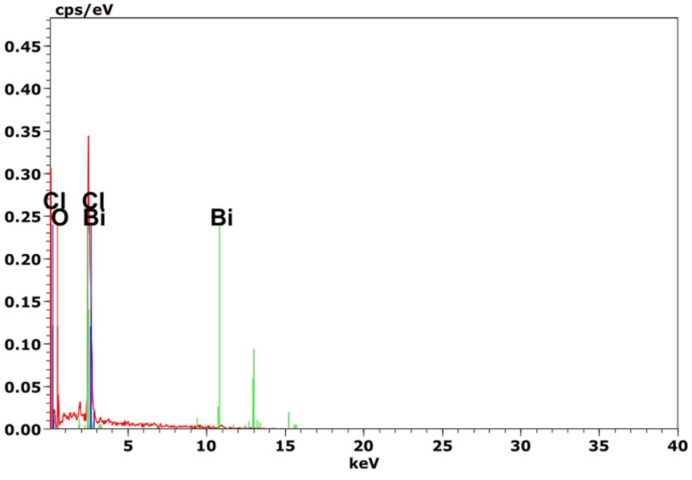
EDS of BC-1.5.

The crystal phase of the as-obtained samples is further confirmed by X-ray diffraction. Figure 3 is an X-ray diffraction pattern of as-prepared samples. It can be seen that the diffraction peak of the XRD pattern of pure β-Bi_2_O_3_ at 2*θ* positions of 27.95°, 31.76°, 32.69°, 46.22°,46.90°, 54.27° is consistent with that of tetragonal phase of the Bi_2_O_3_ (JCPDS card no. 27–0050). The sharp peaks of the XRD pattern of pure BiOCl at 2*θ* positions of 11.78°, 25.72°, 32.45°, 33.26° is consistent with that of the tetragonal phase of BiOCl (JCPDS card No. 06–0249). Moreover, no other diffraction peaks appeared, indicating that the prepared β-Bi_2_O_3_ and BiOCl have higher purity. From the XRD patterns of the β-Bi_2_O_3_/BiOCl composites with different ratios of β-Bi_2_O_3_ and BiOCl, it can be clearly seen that the characteristic peaks of the β-Bi_2_O_3_ and BiOCl appear in the diffraction peaks of these heterojunction photocatalysts. With the increase of the amount of HCl, the intensity of the β-Bi_2_O_3_ characteristic peak decreases significantly, but the intensity of the BiOCl diffraction peak is significantly enhanced at the same time, this is consistent with the SEM test results. No other diffraction peaks are observed, which indicates there are no other impurities in the samples. In order to determine the elemental composition, BC-1.5 was examined by EDS attached to SEM. As shown in Figure 4, the main elements of as-prepared materials are Bi, O, and Cl, which indirectly means the sample is β-Bi_2_O_3_/BiOCl. Moreover, there are no other elements in the EDS spectra, which indicates that the BC-1.5 is pure.

**Figure 5:**
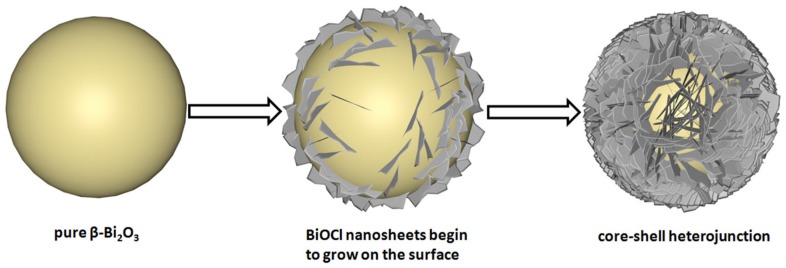
Schematic illustration of the synthesis of β-Bi_2_O_3_/BiOCl core-shell heterojunction.

**Figure 6:**
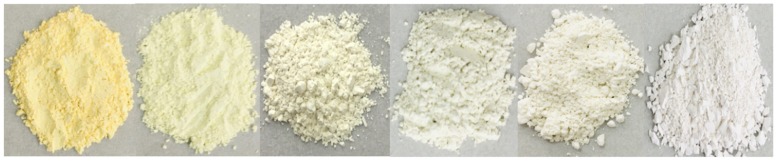
The color of each sample; from left to right are β-Bi_2_O_3_, BC-0.5, BC-1, BC-1.5, BC-1.8, and BiOCl.

Based on the above phase composition and microstructure analysis of the samples, the schematic formation illustration of β-Bi_2_O_3_/BiOCl is described in Figure 5. First, Bi_2_O_3_ is gradually dissolved by HCl to form intermediate product BiO^+^. Second, BiO^+^ quickly combines with Cl^−^ to form a BiOCl precipitate that grows on the surface of the sphere. Finally, With the increase of HCl concentration, more and more BiOCl nanosheets were formed, and finally, flower spherical core-shell heterojunction was formed. The photographic image (Figure 6) showed the color of each sample having different ratios. The color of the β-Bi_2_O_3_ is pale yellow. As the amount of HCl added gradually increased, the number of BiOCl on the β-Bi_2_O_3_ surface gradually increased, so that the yellow color gradually becomes lighter. Finally, pure BiOCl is white. This is consistent with the results discussed above.

### 3.2. XPS Analysis of the Samples

**Figure 7:**
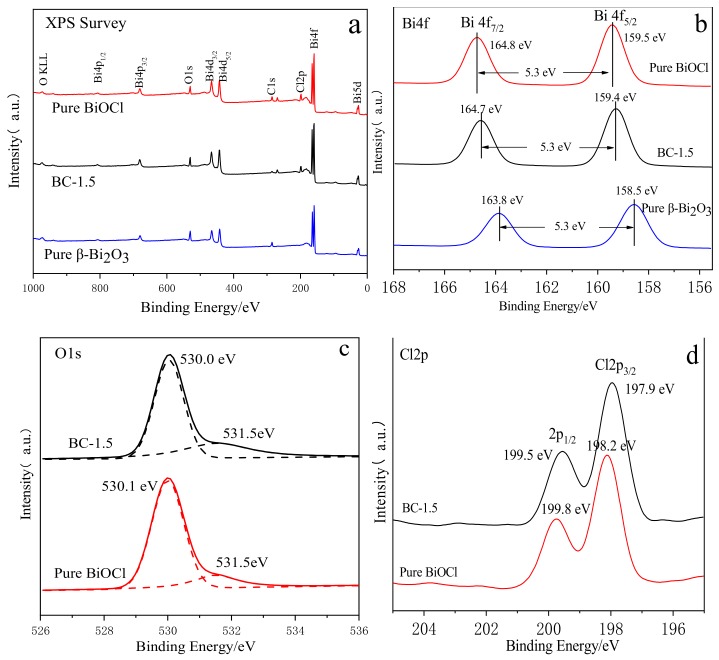
(**a**) XPS survey spectra of β-Bi_2_O_3_, BiOCl, and BC-1.5. (**b**) High-resolution Bi 4f XPS spectra of β-Bi_2_O_3_, BiOCl, and BC-1.5. (**c**) High-resolution O 1s XPS spectra and simulated Gaussian line shapes of BiOCl, and BC-1.5. (**d**) High-resolution Cl 2p XPS spectra of β-Bi_2_O_3_, BiOCl, and BC-1.5.

The elemental composition and surface chemical state of the samples were determined by X-ray photoelectron spectroscopy. The binding energy positions of all the peaks in Figure 7 were corrected using C 1s’s standard binding energy of 284.6 eV. As can be seen from Figure 7a, in the survey spectrum of BC-1.5 sample, the characteristic peaks of the four elements are detected, corresponding to the four elements Bi, O, Cl and C, respectively, wherein the peak of C 1s is derived from the test instrument itself. Figure 7b,d are high-resolution XPS spectrum of the Bi 4f, O 1s, and Cl 2p characteristic peaks of the samples, respectively. For β-Bi_2_O_3_, the spectrum of Bi 4f at the binding energies at 158.5 eV and 163.8 eV correspond to the Bi 4f_5/2_ and Bi 4f_7/2_ levels, respectively, which correspond to Bi^3+^ [39,42]. For the pure BiOCl sample, the binding energies corresponding to the Bi 4f_5/2_ and Bi 4f_7/2_ energy levels are located at 159.5 eV and 164.8 eV, respectively, which are assigned to Bi^3+^ in BiOCl. For the BC-1.5 sample, the value of the peak separation was the same as β-Bi_2_O_3_ and BiOCl. However, compared with the pure β-Bi_2_O_3_ peak of Bi 4f, the binding energy position of the Bi 4f peak in the BC-1.5 sample slightly shifted toward higher values, indicating that there is a strong interaction between β-Bi_2_O_3_ and BiOCl. The signal peak of O 1s in the sample is shown in Figure 7c. After deconvolution, it can be seen that O 1s of these two materials have two peak positions, which are around 530.1 eV and 531.5 eV, respectively. The peak at which the binding energy is located at 530.1 eV indicates the presence of lattice oxygen, which shows that the bonding properties of the lattice oxygen have not changed during the change of the material. The peak position around 531.5 eV corresponds to the binding energy of the adsorbed oxygen on the surface of the material. As can be seen from Figure 7c, the proportion of oxygen adsorbed on the surface of BC-1.5 is higher than that of BiOCl. Adsorbed oxygen is a kind of active and strong oxidizing group, which can promote the photocatalytic oxidation reaction. Figure 7d demonstrates that the Cl element in the samples is in the form of Cl^−^. The peaks at the binding energies at 197.9 eV (or 198.2 eV) and 199.5 eV (199.8 eV) correspond to the Cl 2p_3/2_ energy level and the 2p_1/2_ energy level [43], respectively. It can be seen that after etching β-Bi_2_O_3_ with HCl, the binding energy position of the Cl 2p peak shifts toward the low energy direction. These results further demonstrate the simultaneous presence of β-Bi_2_O_3_ and BiOCl in the β-Bi_2_O_3_/BiOCl heterojunction, and there is a strong interaction between them, which accelerates the transfer of photogenerated charges and thus increases the photocatalytic activity.

### 3.3. BET Analysis of the Samples

**Figure 8:**
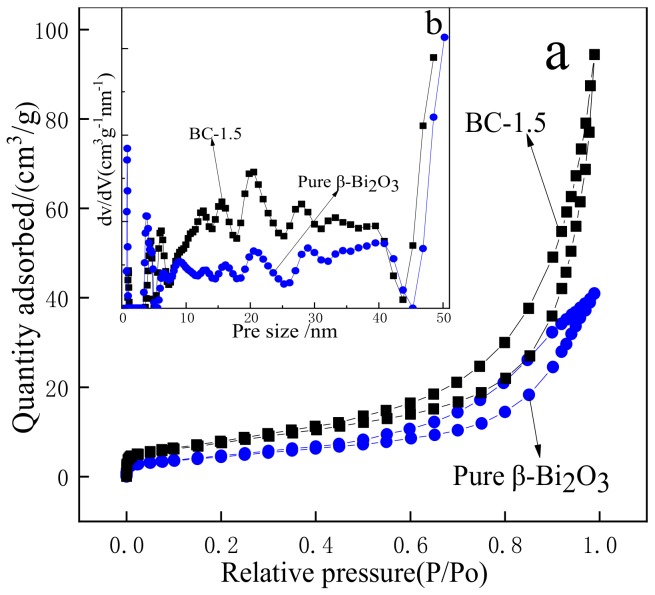
Nitrogen adsorption-desorption isotherms (**a**) and the pore size distribution of samples (**b**).

The specific surface area can greatly affect the exposure of the active site of the catalyst and is, therefore, an important factor affecting the catalytic activity (Figure 8) are the nitrogen adsorption-desorption isotherms (a) and corresponding pore size distribution curves (b) of β-Bi_2_O_3_ and BC-1.5. As shown in Figure 8a, the isotherms of samples all belong to type IV cure with H3 hysteresis loops at relative pressures ranging from 0.45 and 1.0, which reveals that β-Bi_2_O_3_ and BC-1.5 are classified into mesoporous materials, the pore size distribution curve is shown in the inset also verifies this. As can be seen from Figure 8b, the pore size of β-Bi_2_O_3_ and BC-1.5 were mainly distributed in 5–40 nm, and the proportion of BC-1.5 was more than β-Bi_2_O_3_. The specific surface areas of β-Bi_2_O_3_ and BC-1.5 were calculated by BET to be 17.026 m^2^/g and 27.711 m^2^/g, respectively. The increase in the specific surface area of β-Bi_2_O_3_ after HCl etching may be related to the growth of many BiOCl nanosheets on the surface. In the field of photocatalysis, a large specific surface area facilitates the adsorption of more reactants on the surface of the catalyst, and a higher pore volume facilitates the rapid diffusion of various reactants and products during the photocatalytic reaction, thereby, improving the photocatalytic activity and degrading organics more quickly. So characterizing the specific surface area and pore size distribution of the photocatalyst is advantageous for studying the photocatalytic properties of the material [44]. For the degradation of TC, the photocatalytic activity of BC-1.5 may be higher than that of β-Bi_2_O_3_ because of its large specific surface area and a higher proportion of pores.

### 3.4. UV-Vis Absorption Spectrum Analysis of the Samples

**Figure 9:**
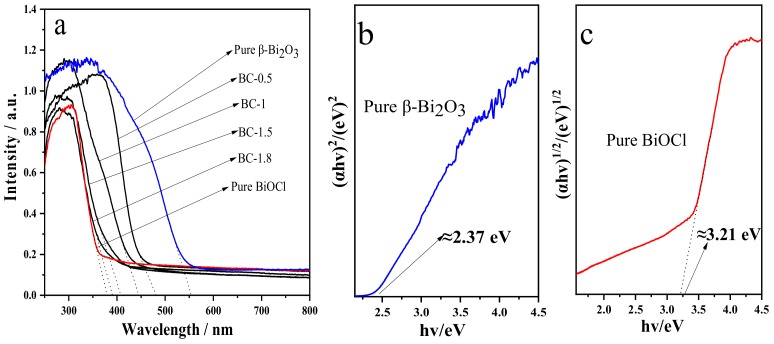
UV-vis diffuse reflectance spectra (**a**), calculated energy bandgap of β-Bi_2_O_3_ (**b**), and BiOCl (**c**).

In order to investigate the optical absorption properties of the samples, we used UV-visible diffuse reflectance spectroscopy to characterize β-Bi_2_O_3_, BiOCl, and different proportions of β-Bi_2_O_3_/BiOCl samples, the results are shown in Figure 9. As can be seen from Figure 9a, β-Bi_2_O_3_ has strong absorption in the visible light region, and its absorption edge is about 550 nm. However, BiOCl only absorbs in the UV-light region, and the absorption edge is about 378 nm. The absorption edge of β-Bi_2_O_3_/BiOCl samples is between β-Bi_2_O_3_ and BiOCl. With the concentration of HCl increases, the absorption edge of the sample gradually approaches BiOCl. Therefore, according to Beer–Lambert’s law, the ratio of β-Bi_2_O_3_ and BiOCl for different samples can be estimated by the value of the absorption edge. The results are very close to the theoretical values.

In addition, the forbidden bandwidth of a semiconductor can be estimated according to the following Equation (3):

(*αhν*) = *A* (*hν* − *Eg*)^*n*/2^ (3)

In this formula, *α*, *h*, *v*, *Eg*, and *A* are the optical absorption coefficient, Planck constant, photon frequency, band gap energy, and proportional constant. The value of the coefficient *n* depends on the characteristics of the semiconductor, *n* = 1 for a direct bandgap semiconductor, and *n* = 4 for an indirect bandgap semiconductor. β-Bi_2_O_3_ is a direct bandgap semiconductor, so *n* = 1, and BiOCl is an indirect bandgap semiconductor, so *n* = 4. The forbidden bandwidths of β-Bi_2_O_3_ and BiOCl can be plotted with energy (*hv*) by (*αhv*)^2^ and (*αhv*)^1/2^, respectively. The obtained straight-line segment of the graphic is extended to the x-axis, and the corresponding values at the intersection point are the forbidden bandwidths of β-Bi_2_O_3_ and BiOCl. According to Figure 9b,c, it is estimated that the forbidden bandwidth of β-Bi_2_O_3_ is about 2.37 eV, which is similar to the reported value [45], and the forbidden bandwidth of BiOCl is about 3.21 eV, which is similar to the results reported in the literature [39,46].

### 3.5. Analysis of Photocatalytic Properties

**Figure 10:**
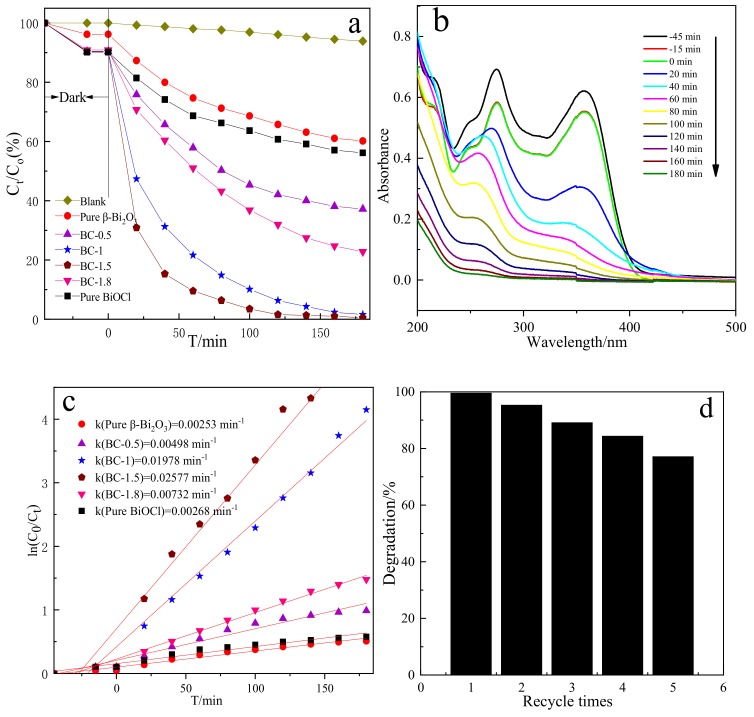
(**a**) Photodegradation of TC by different photocatalysts. (**b**) UV-visible absorption spectra for the degradation of TC using the BC-1.5. (**c**) The ln(C_0_/C_t_) versus time curves of the photodegradation of TC. (**d**) The repeatability tests on the BC-1.5 photocatalyst for five cycles.

The photocatalytic properties of β-Bi_2_O_3_ and its composites were investigated by degrading TC under visible light. Figure 10a shows the degree of degradation of TC by different samples. From the degradation curve, we can see that the catalytic effects of different composite samples are different. The results show that TC is relatively stable in the absence of a catalyst, eliminating the effect of light on the TC solution. As can be seen from Figure 10a, compared with the β-Bi_2_O_3_ and BiOCl photocatalysts, the photocatalytic activity of the composite photocatalyst was significantly enhanced. With the increase of BiOCl on the surface of β-Bi_2_O_3_, its catalytic activity first increased and then decreased. When the dosage of HCl is 3.225 mL, the catalytic effect is the best; the degradation rate exceeded 99.5% after 180 min of visible light irradiation. However, light is refracted back and forth between the nanosheets, part of the light is directly reflected, and part of the light is blocked. Therefore, the more nanosheets, the more light is reflected and blocked, and the less light β-Bi_2_O_3_ is exposed to. At the same time, due to the large reduction in β-Bi_2_O_3_, the interface between β-Bi_2_O_3_ and BiOCl decreases. These factors together lead to a decline in photocatalytic performance. Figure 10b is a UV-visible absorption spectrum of the process in which the BC-1.5 sample degrades the TC solution. Under the irradiation of visible light, the intensity of the maximum characteristic absorption peak (λ = 356 nm) of the TC solution gradually decreases with the increase of the illumination time. From the curve, we can see that when the time reaches 180 min, the characteristic peak almost disappeared, indicating that the sample has a good ability to degrade the TC solution photo-catalytically.

Figure 10c shows the kinetic linear fitting effect of photocatalytic degradation of TC in different samples. It can be seen from the figure that the linear relationship between ln (C_0_/C_t_) and t is good, indicating that the reaction of the composite photocatalyst to degrade TC is a pseudo-first-order model reaction, and the photocatalytic reaction conforms to the Langmuir–Hinshelwood kinetic equation k is the apparent rate constant of the reaction, as can be seen from the value of k in Figure 10c, the reaction rate of all composites are faster than that of β-Bi_2_O_3_ and BiOCl, and the corresponding photocatalytic performance is improved. BC-1.5 has the highest degradation rate (k = 0.02577), and its photocatalytic activity was 10.2 and 9.6 times that of β-Bi_2_O_3_ and BiOCl, respectively.

The cycling ability and stability of the photocatalyst is of extreme significance for practical application. To evaluate the performance of the composite catalyst, the recycling experiment is performed for the photocatalytic degradation of TC by the BC-1.5 in Figure 10d. As shown in Figure 10d, the degradation rate of TC is 99.5% after the first used of BC-1.5. After washing and drying, BC-1.5 is secondly used, and the degradation rate of TC is 95.3%, the degradation rate declines by 4.2%. This may be due to the fact that adsorbed TC on BC-1.5 is difficult to wash completely during the process of regeneration. After five consecutive recycling, the degradation rate of TC still keeps at about 77.1%, which declines by 22.4% than that of the first time. There was no significant decrease in photocatalytic performance under the slight loss of material during the recovery process. These results indicate that the BC-1.5 has good photocatalytic stability, and the as-prepared heterojunction photocatalyst has a promising used in the field of wastewater treatment.

### 3.6. Photodegradation Mechanism of the Samples

**Figure 11:**
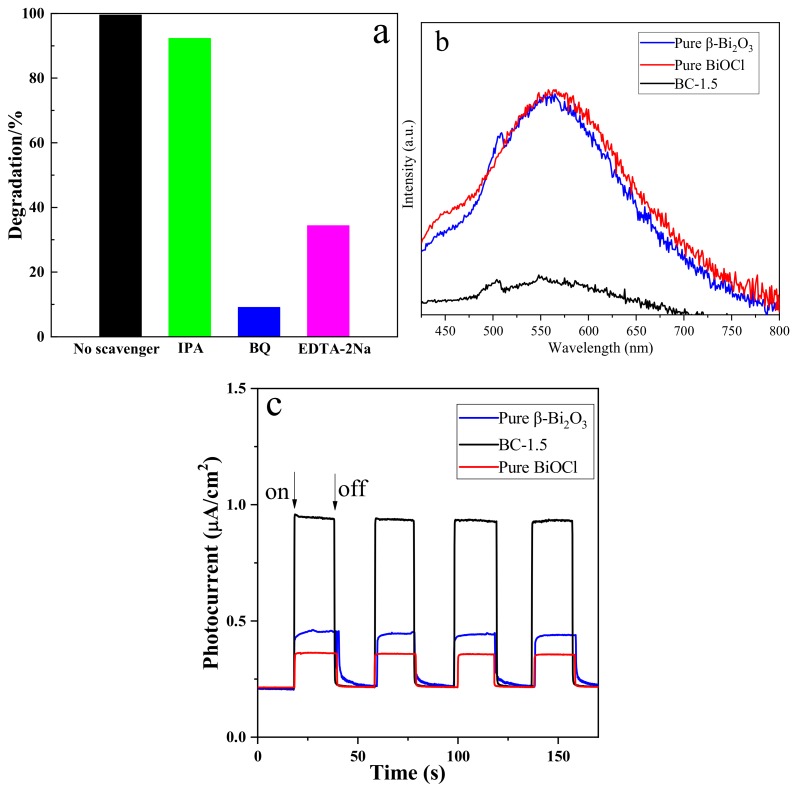
(**a**) Active species trapping experiments of BC-1.5 for TC photodegradation. (**b**) Photoluminescence spectra of β-Bi_2_O_3_ and BiOCl, β-Bi_2_O_3_/BiOCl heterojunctions (BC-1.5). (**c**) Transient photocurrent responses of β-Bi_2_O_3_, BiOCl, and BC-1.5.

In order to further study the photocatalytic mechanism and explore the influence of different active species on the photocatalytic activity, we carried out a free radical capture experiment. Isopropyl alcohol (IPA, 10 mmol/L), ethylenediaminetetraacetic acid disodium salt (EDTA-2Na, 5 mmol/L) and p-benzoquinone (BQ, 1 mmol/L) are used as trapping agents for hydroxyl radicals (•OH), holes (h^+^) and superoxide radicals(•O_2_^−^), respectively. The addition of different capture agents has different effects on the degradation of TC by BC-1.5 sample under visible light. As shown in Figure 11a, it can be seen from the corresponding degradation rate that in the presence of BQ and EDTA-2Na, the degradation rate of TC was significantly reduced, and the presence of BQ reduced the degradation rate most (from 99.5% to 9.1%), while the presence of IPA has little effect on the degradation rate. From the above results, it can be concluded that •O_2_^−^ plays a key role in the photocatalytic degradation process. When EDTA-2Na was added, the TC photodegradation was also decreased by 34.3%, indicating that h^+^ was also the main species. However, it can be seen from the figure that the effect of •OH is very small.

The activity of the photocatalyst is related to the absorption range of light, the absorption intensity, and the separation of photogenerated electrons and holes. Photoluminescence (PL) is a simple technique for studying the optical properties of semiconductor materials. Studies have shown that the fluorescence intensity is related to the recombination rate of electrons and holes [47]. The stronger the intensity, the higher the recombination rate. Figure 11b shows the fluorescence spectrum of β-Bi_2_O_3_, BiOCl, and BC-1.5. The excitation wavelength during the test was set to 340 nm. It can be seen that the fluorescence intensity of the BC-1.5 composite sample is significantly lower than that of β-Bi_2_O_3_ and BiOCl, indicating that the β-Bi_2_O_3_/BiOCl heterojunction can effectively reduce the recombination rate of electrons and holes and has high photocatalytic efficiency. At the same time, this is consistent with the fact that the performance of BC-1.5 is obviously better than that of β-Bi_2_O_3_ and BiOCl in the photocatalytic experiment. The separation efficiency of photogenerated electrons and holes of the photocatalyst can also be characterized by electrochemical methods. Figure 11c is a transient photocurrent response curve of the samples under the illumination of the 300 W Xe lamp. The interval between each time the light is turned on and off is 20 s. It can be seen that the photocurrent response intensity of the BC-1.5 composite sample is significantly increased compared with β-Bi_2_O_3_ and BiOCl. Generally, the larger the photocurrent density, the stronger the separation ability of electrons and holes, which is beneficial to increase the photocatalytic activity. The β-Bi_2_O_3_/BiOCl heterojunction exhibits a strong photocurrent response intensity probably due to its larger localized microdomain, which facilitates the separation and transmission of e^−^/h^+^ pairs.

The band positions of the photocatalyst have a great influence on the separation and transformation of e^−^/h^+^ pairs. The positions of the valence band (VB) and the conduction band (CB) of β-Bi_2_O_3_ and BiOCl can be calculated by empirical equations:

E_CB_ = X − E_c_ − 0.5 Eg (4)

E_VB_ = E_CB_ + Eg (5)

where X is the absolute electronegativity of the semiconductor (the geometric mean of the constituent atoms), E_VB_ is the position of the edge of the valence band, E_CB_ is the position of the edge of the conduction band, Eg is the bandgap of the semiconductor, and E_c_ is the energy of the free-electron of hydrogen (about 4.5 eV). The X values of β-Bi_2_O_3_ and BiOCl are 5.95 eV and 5.0 eV, respectively. It is estimated by the results of the DRS test that the band gaps of β-Bi_2_O_3_ and BiOCl are 2.37 eV and 3.21 eV, respectively. Therefore, the conduction band positions of β-Bi_2_O_3_ and BiOCl are 0.27 eV and−1.11 eV, respectively, and the valence band positions are 2.64 eV and 2.10 eV, respectively. Based on the above results, it was found that the n-type β-Bi_2_O_3_ and p-type BiOCl can form a good B-type heterojunction.

**Figure 12:**
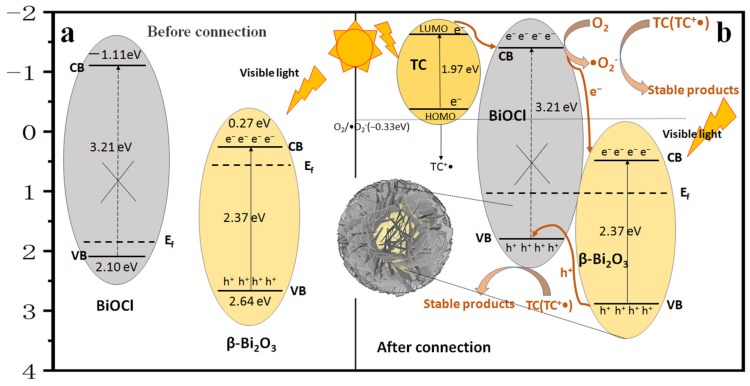
Possible photocatalytic mechanism of TC degradation by β-Bi_2_O_3_/BiOCl under visible light irradiations. (**a**) Before connection. (**b**) After connection.

Based on the discussion of the above experimental results, the photocatalytic activity of the composite catalyst sample was enhanced mainly because a p-n type heterojunction is formed between β-Bi_2_O_3_ and BiOCl. Figure 12 shows the photocatalytic mechanism of the degradation of TC by composite catalyst sample under visible light. For the n-type β-Bi_2_O_3_, its Fermi level is closer to the conduction band, and for the p-type BiOCl, its Fermi level is closer to the valence band. When β-Bi_2_O_3_ and BiOCl are connected together, electrons diffuse from the Bi_2_O_3_ to the BiOCl, and holes diffuse in the opposite direction from the BiOCl to the Bi_2_O_3_. Diffusion results in the accumulation of positive charges in the heterojunction Bi_2_O_3_ semiconductor region, while the accumulation of negative charges near the heterojunction BiOCl semiconductor region. When the BiOCl semiconductor and the Bi_2_O_3_ semiconductor Fermi level reach equilibrium, a built-in electric field from the Bi_2_O_3_ semiconductor to the BiOCl semiconductor is formed, and the internal electric field prevents the charge from continuing to diffuse between the two semiconductors. At the same time, the energy bands of the two semiconductors move in opposite directions as a whole until the Fermi level of the two semiconductors is flat, and finally, a built-in electric field is generated at the p-n junction interface from Bi_2_O_3_ to BiOCl. The position of the photocatalyst band has an important influence on the generation and separation of photoinduced e^−^/h^+^ pairs. Moreover, the transfer route of photogenerated electrons and holes can be explained by the conduction band minimum of the photocatalyst and the highest value of the valence band. After the heterojunction is formed, the position of the BiOCl conduction band is more negative than the position of the β-Bi_2_O_3_ conduction band, and the position of the β-Bi_2_O_3_ valence band is more positive than the position of the BiOCl valence band. Hence, this system is a kind of B-type heterojunction. When the β-Bi_2_O_3_/BiOCl sample is irradiated with visible light since β-Bi_2_O_3_ has a narrow bandgap (2.37 eV), β-Bi_2_O_3_ can be excited by visible light to generate photogenerated e^−^ and h^+^, and the e^−^ in the VB of β-Bi_2_O_3_ is excited to its CB, so h^+^ is produced on the VB of β-Bi_2_O_3_. However, BiOCl could not be excited by visible light due to the large bandgap of BiOCl (Figure 9c). According to previous reports, TC molecules show a small energy gap (1.97eV) between HOMO and LUMO [48,49]. Similar to photosensitization, TC molecules absorbed on the surface of photocatalysts are excited by visible light to produce e^−^ and the e^−^ transfer to the CB of BiOCl crystallites due to the more negative potential of TC lowest unoccupied molecular orbital (LUMO) level. The excited TC molecule becomes an unstable 1-electron oxidation product (TC^+^•) of the TC. Since the CB boundary position of BiOCl is more negative than that of β-Bi_2_O_3_, the e^−^ on the BiOCl surface can easily pass through the two-phase interface to reach the CB of β-Bi_2_O_3_. Similarly, because of the difference in VB positions of the two phases, h^+^ generated on the VB of the β-Bi_2_O_3_ are also transferred to the VB of BiOCl, forming an internal electric field across the interface. Therefore, the recombination of photoinduced e^−^/h^+^ pairs in the β-Bi_2_O_3_/BiOCl photocatalyst is suppressed.

Capture experiments have shown that •O_2_^−^ and h^+^ are the main active species in the degradation process. The e^−^ that migrated to the CB of BiOCl can react with dissolved O_2_ to generate •O_2_^−^ due to more negative CB potential than that of O_2_/•O_2_^−^ (−0.33eV). But the e^−^ generated on the β-Bi_2_O_3_ conduction band and transferred from BiOCl cannot react with dissolved O_2_ to generate •O_2_^−^ due to more positive CB potential than that of O_2_/•O_2_^−^. The h^+^ generated by β-Bi_2_O_3_ accumulate on the surface of the BiOCl valence band. The •O_2_^−^ and h^+^ then react with the toxic organics (TC and TC^+^•) in the solution to degrade them into stable products. The •O_2_^−^, h^+^, and e^−^ can react with the adsorbed water (or hydroxide ions) to form a strong oxidant •OH, •OH can degrade the adsorbed TC into stable products. However, since •OH is not the main active species in the photocatalytic reaction of TC on β-Bi_2_O_3_/BiOCl, the reaction with •OH does not constitute a major step. Previous research has also proved this [49]. In the process of photocatalytic degradation of TC, the possible reaction process is as follows:

TC + hv (λ > 400 nm) → TC’(6)

TC’ + β-Bi_2_O_3_/BiOCl → TC^+^• + β-Bi_2_O_3_/BiOCl(e^−^) (7)

β-Bi_2_O_3_/BiOCl + hv → e^−^ + h^+^ (8)

O_2_ + e^−^ (BiOCl_cb_) → •O_2_^−^ (9)

TC(TC^+^•) + •O_2_^−^(BiOCl_cb_) → Degradation products (10)

TC(TC^+^•) + h^+^ (BiOCl_vb_) → Degradation products (11)

Not the main step:

h^+^ + OH^−^ → •OH (12)

•O_2_^−^ + 2H^+^ → H_2_O_2_ (13)

H_2_O_2_ + e^−^ → OH^−^ + •OH (14)

TC(TC^+^•) + •OH → Degradation products (15)

Combined with the above discussion and similar reports, we conclude that BiOCl plays a major role in the β-Bi_2_O_3_/BiOCl heterojunction photocatalytic reaction, and the presence of β-Bi_2_O_3_ causes a heterojunction electric field to form at the interface between β-Bi_2_O_3_ and BiOCl. This electric field accelerates the separation of e^−^/h^+^ pairs and inhibits their recombination. At the same time, the presence of β-Bi_2_O_3_ effectively broadens the absorption range of the β-Bi_2_O_3_/BiOCl heterojunction photocatalyst for visible light. Therefore, in the process of visible light degradation of organic wastewater, β-Bi_2_O_3_/BiOCl heterojunction has higher photocatalytic activity than β-Bi_2_O_3_ and BiOCl. In addition, the BiOCl nanosheets are located outside of the β-Bi_2_O_3_/BiOCl heterojunction photocatalyst, so that the effective catalytic active sites are not obscured by the composite structure. β-Bi_2_O_3_/BiOCl composite materials are harmless to the environment, the synthesis process is simple, the manufacturing cost is low, and mass production is possible. Therefore, the novel heterojunction visible light photocatalyst has broad application prospects in the field of organic wastewater treatment.

## 4. Conclusions

In summary, β-Bi_2_O_3_/BiOCl heterojunction photocatalyst was successfully synthesized by ultrasound-assisted hydrochloric acid impregnation method, which is simple and low cost. Compared with β-Bi_2_O_3_ and BiOCl, all β-Bi_2_O_3_/BiOCl heterojunction photocatalysts displayed remarkably enhanced visible photocatalytic activities in degrading tetracycline hydrochloride (TC). The increase in photocatalytic activity is due to the formation of a heterojunction between β-Bi_2_O_3_ and BiOCl, which effectively promotes the separation of photogenerated e^−^/h^+^ pairs. The results showed that the reaction of the composite photocatalyst to degrade TC is a pseudo-first-order model reaction, and the photocatalytic reaction conforms to the Langmuir-Hinshelwood kinetic equation. Moreover, the removal rate of β-Bi_2_O_3_/BiOCl heterojunction for TC still keeps at a very high degree after five consecutive cycles, which demonstrates the outstanding photocatalytic stability of as-obtained samples. Additionally, the capture experiments have shown that •O_2_^−^ and h^+^ are the main active species in the degradation process, and we conclude that BiOCl plays a major role in the β-Bi_2_O_3_/BiOCl heterojunction photocatalytic reaction. However, the presence of β-Bi_2_O_3_ causes a heterojunction electric field to form at the interface between β-Bi_2_O_3_ and BiOCl. Therefore, β-Bi_2_O_3_/BiOCl has a promising candidate as a competitive photocatalyst in wastewater treatment, and this paper provides assistance for studying the photocatalytic mechanism of heterojunction photocatalyst.
